# Sighted and visually impaired students’ perspectives of illustrations, diagrams and drawings in school science

**DOI:** 10.12688/wellcomeopenres.9968.1

**Published:** 2016-11-15

**Authors:** Celia McDonald, Susan Rodrigues

**Affiliations:** 1 Liverpool Hope University, Liverpool, UK

**Keywords:** Diagrams, Illustrations, School science, Visually impaired, School students

## Abstract

**Background**: In this paper we report on the views of students with and without visual impairments on the use of illustrations, diagrams and drawings (IDD) in science lessons.

**Method**: Our findings are based on data gathered through a brief questionnaire completed by a convenience sample of students prior to trialling new resource material. The questionnaire sought to understand the students’ views about using IDD in science lessons. The classes involved in the study included one class from a primary school, five classes from a secondary school and one class from a school for visually impaired students.

**Results**: Approximately 20% of the participants thought that the diagrams were boring and just under half (48%) of the total sample (regardless of whether they were sighted or visually impaired) did not think diagrams were easy to use. Only 14% of the participants felt that repeated encounters with the same diagrams made the diagrams easy to understand. Unlike sighted students who can ‘flit’ across diagrams, a visually impaired student may only see or touch a small part of the diagram at a time so for them ‘fliting’ could result in loss of orientation with the diagram.

**Conclusions**: Treating sighted and visually impaired pupils equally is different to treating them identically. Sighted students incidentally learn how to interpret visual information from a young age. Students who acquire sight loss need to learn the different rules associated with reading tactile diagrams, or large print and those who are congenitally blind do not have visual memories to rely upon.

## Introduction

A project funded by the Wellcome Trust to develop educational resources for visually impaired students has enabled us to collect data that explore how visually impaired students and sighted students currently use and make sense of science diagrams in school classrooms. Most of the topics in school science education include illustrations, diagrams and drawings (IDD) which accompany text and are intended to help learners make better sense of key science concepts. However, Bergey *et al.* (2015) reported that pupils in general have difficulty understanding science diagrams. Similarly, Treagust & Tsui (2013) suggested that coordinating multiple representations (by switching between representations) is a key skill requirement in underlying diagram comprehension in science. Some studies have explored whether diagram comprehension can be improved through training. For example, Bartholomé & Bromme (2009) suggested adding hyperlinks. Bergey *et al.* (2015) identified other studies reporting on the impact of instructing school students about the conventional and common features found in IDD.

Agarwal *et al*. (2014) suggested that the issues identified as challenging for sighted pupils are exacerbated for students with visual impairments. Students with visual impairments may find concepts in science, technology, engineering and mathematics (STEM) subjects even more difficult to grasp because STEM subjects include concepts that rely heavily on visual representation in the form of diagrams, graphs and charts. Yet, as Bergey *et al*. (2015) reported common tasks in many school science text books require students to coordinate texts and IDD. Unfortunately, research shows that students often do not pay attention to IDD while reading science texts in schools ( Cromley *et al*., 2010). The Ainsworth (2006) model of learning with representations identified the role of tasks. A task refers to what the student has to do with the IDD, bearing in mind familiarity with the IDD style, topic, spatial skill, working memory capacity and the challenge set ( Khooshabeh & Hegarty, 2010).

Several researchers have reported on the skills required to understand diagrams:

1) Kress & Van Leeuwen (1996) reported on the grammar of visual design, which recognizes that images have rules like languages.
2) Gilbert *et al*. (2008) discussed “metavisual capability” with regard to diagrams in science education and they highlighted the importance of visual literacy and interpretive codes when relating a diagram to a phenomenon.
3) Unsworth (2001) discussed how representational structures visually construct the nature of an event, the circumstances in which they occur and the relationship between participants and objects. Unsworth (2001) explained how visual resources help construct a relationship between the viewer and what is viewed.

Kumar *et al*. (2001) suggested that visually impaired students may not have a lower cognitive ability than their sighted peers, but because schools rely heavily on vision, visually impaired students experience academic problems. Kumar *et al*. found that “overcoming barriers to experiencing activities that are unfamiliar is critical in stimulating the intellectual growth of students with visual disabilities”.

Research reporting on improving diagram comprehension tactics through, for example, the use of hyperlinks ( Bartholomé & Bromme, 2009) or learner constructed diagrams ( Leopold & Leutner, 2012), or the teaching of conventional features of diagrams ( Cromley *et al*., 2013), uses approaches with sighted pupils to evaluate the impact of these tactics. But these strategies might not be appropriate in exploring how the visually impaired student makes sense of science diagrams. For example, Cromley *et al*. (2013) report on a 25-item measure of diagrams comprehension in biology in which the use of multiple-choice items includes a colour key and invites the students to identify what this colour key indicates about an animal’s features. For some visually impaired students the question focus on colour renders the multiple-choice items inappropriate.

While researchers signal the importance of IDD in science they also signal the need to be able to understand the rules associated with IDD or codes implicit within IDD in order to be able to reach the best interpretation of an IDD. These are visual rules and visual experiences. In order for visually impaired students to have a better understanding of diagrammatic rules or codes when using IDD in science lessons, they need to be encouraged at an early age, not only to learn haptically with explanation, but some advocate that they also need to learn to draw. As Maneki (2013) explained, to be tactile fluent the person needs to be proficient in Braille reading and writing and proficient in drawing and interpreting diagrams. Maneki (2013) suggests that visually impaired children, like sighted children, should be encouraged to read and draw before kindergarten. This needs to be supported throughout their education, just as sighted students are supported in their learning through experience (including observation and making drawings).

This would tie in with Constructivist views on learning, which suggest that a learner responds to sensory experiences by building personal cognitive structures, which constitute the meaning and understanding of their world ( Saunders, 1992). As Harlen (2010) noted, babies move their heads and eyes looking particularly at straight lines and contrasts and soon learn to predict movement. For example, when a ball rolls behind a screen they look at the point where it should reappear. In the same way children learn at a very early age about how something is visibly obscured when something is in front of it. These are aspects of understanding science and drawings/diagrams at an early age. However, if a student is congenitally blind or has (some form of) congenitally partial sight then many of the aspects listed earlier are not incidentally learned. This lack of incidental learning means that students with a visual impairment are at a disadvantage when it comes to understanding science diagrams. Similarly, the ability to understand IDD can also be influenced by the student’s life experiences.

## Project synopsis

In this article we report on the views of sighted and visually impaired students when they encounter various IDD in science lessons. We use the Ponchillia & Ponchillia (1996) view of visual impairment, i.e. any degree of vision loss, including total blindness that affects an individual's ability to perform the tasks of daily life. This may include some of the following: low vision, blindness, congenital birth, adventitiously blind and light perception. We are interested in how visually impaired learners construct meaning and understanding of science concepts when responding to experiences that involve the use of science IDD. The first step for us in addressing this interest was to ascertain the views of sighted and visually impaired students with regard to the current use of IDD in their classes.

## Methodology

We identified and contacted schools with students with visual impairments (VIP) through formal routes (through the University’s Special Educational Needs (SEN) contact, the University’s partner schools and through rehabilitation officers at three local authorities). Our research sample was therefore one of convenience. Ethics approval for the research project was given by the Faculty of Education at Liverpool Hope University.

The sample included six mainstream school classes (one primary and five secondary) and a secondary class in a school for visually impaired students. As this was a convenience volunteer sample, the nature of the lesson and the topic being taught were not within our control. (In England, year 5 refers to 9–10 year old students, year 8 refers to 12–13 year old students, year 9 refers to 13–14 year old students, year 10 refers to 14–15 year old students, year 11 refers to 15–16 year old students ( Gov.UK, 2016)).

- Mainstream primary class A involved 26 year 5 students, (including 1 VIP student) learning about the phases of the moon.
- School for the visually impaired class B involved 4 (all VIP) year 8 students recapping acids and alkalis, digestive system, electrolysis and distillation.
- Mainstream secondary school class C involved 14 lower ability year 8 students (including 1 VIP student).
- Mainstream secondary school class D involved 24 year 9 students (including 1 VIP student) completing a GCSE physics topic and starting on GCSE biology topic.
- Mainstream secondary school class E involved 20 year 9 (including 1 VIP student) learning about cells.
- Mainstream secondary school class F involved 24 year 10 students (including 1 VIP student) learning about how the universe was formed.
- Mainstream secondary school class G involved 9 year 11 students (including 1 VIP student) learning about kinetic and gravitational energy.

Consent: The schools taking part in the study acted ‘in loco parentis’, thereby meeting the University’s Ethics policy. Prior to issuing the student questionnaire students were asked to complete an informed consent form (versions of the questionnaire and consent form were adapted where necessary to address student access). The form indicated the purpose of our project (trying to improve IDD that are used in school science lessons). The informed consent form indicated that we were in the process of developing materials and we wanted the students’ views of the diagrams that they currently use in science lessons. The informed consent form also indicated that completion of the questionnaire was voluntary and that the completed questionnaires would not identify students by name. In order to maintain anonymity, the students placed their questionnaire in a project box in their classroom.

The questionnaire content (see Supplementary material) included 3 questions. Two questions included tick box options and the third question was an open question. The wording used in the first two questions was arrived at after a discussion with two teachers (one with experience in primary school education and one with experience in secondary school education). Hence, for example, the word ‘messy’ rather than ‘cluttered’ was used in question two.

## Results

The tables below present the findings in terms of the number of students (including VIP) who ticked the appropriate box (as a fraction of the total number of students in that class). The table also shows the number of visually impaired (VIP) students in that class who ticked that box (as a fraction of the total number of VIP students in that class). * Please note we use ‘unknown’ in the tables if the VIP student did not identify her/himself when s/he completed the questionnaire.

Our findings show that over 50% of the students in the mainstream secondary classes, years 8 and 9 did not find diagrams easy to understand (57% year 8: 53% year 9; Table 1). In contrast, in the primary school class over 58% stated that they found diagrams easy to understand. Overall, just over half of the total sample (63/120) stated diagrams were easy to use. Given the visually impaired contingent made up less than 10% of the convenience sample, our findings suggest that a significant number of sighted pupils (nearly 50% of our sample) also struggle to understand diagrams in science.

**Table 1.** Students’ response to: “Diagrams in science are easy to understand”.

Class	Year level	Respondent students/Total number of students in class	Respondent VIP students/Total number of VIP students in class
Primary A	5	15/26	1/1
School B	8	3/4	3/4
Secondary C	8	6/14	0/1
Secondary D	9	9/23	1
Secondary E	9	11/20	0/1
Secondary F	10	13/24	unknown *
Secondary G	11	6/9	0/1
**Total**		**63/120**	**5/9**

* Please note we use ‘unknown’ in the tables if the VIP student did not identify her/himself when s/he completed the questionnaire.

Our findings ( Table 2) show that the only visually impaired student who found diagrams boring was in School B. Less than 20% of the total sample found diagrams in science boring.

**Table 2.** Students’ response to: “Diagrams in science are boring to use”

Class	Year level	Respondent students/Total number of students in class	Respondent VIP students/Total number of VIP students in class
Primary A	Year 5	2/26	0/1
School B	Year 8	1/4	1/4
Secondary C	Year 8	0/14	0/1
Secondary D	Year 9	8/23	0/1
Secondary E	Year 9	5/20	0/1
Secondary F	Year 10	6/24	unknown *
Secondary G	Year 11	1/9	0/1
**Total**		**23/120**	**1/9**

* Please note we use ‘unknown’ in the tables if the VIP student did not identify her/himself when s/he completed the questionnaire.

Interestingly (bearing in mind that less than 20% found diagrams ‘boring’), about 25% of the students in mainstream secondary school classes found that the diagrams used in their science lessons were exciting. There was a marked difference between the responses from primary school students and the responses from secondary school students, with 61% of the primary students indicating that they found the diagrams exciting (while two of the four students in the school for visually impaired found diagrams exciting: Table 3.)

**Table 3.** Students’ response to: “Diagrams in science are exciting”

Class	Year level	Respondent students/Total number of students in class	Respondent VIP students/Total number of VIP students in class
Primary A	Year 5	16/26	1/1
School B	Year 8	2/4	2/4
Secondary C	Year 8	3/14	0/1
Secondary D	Year 9	0/23	0/1
Secondary E	Year 9	5/20	1/1
Secondary F	Year 10	4/24	unknown *
Secondary G	Year 11	1/9	0/1
**Total**		**31/120**	**4/9**

* Please note we use ‘unknown’ in the tables if the VIP student did not identify her/himself when s/he completed the questionnaire.

In the school for the visually impaired (School B) 75% of the students found diagrams in science hard work. In the mainstream schools, only the visually impaired students in the lower band found them hard work. Overall, over 50% of our sample of visually impaired students found diagrams hard work, whilst only 23% of the total sample of students found them to be hard work ( Table 4).

**Table 4.** Students’ response to: “Diagrams in science are hard work”

Class	Year level	Respondent students/Total number of students in class	Respondent VIP students/Total number of VIP students in class
Primary A	Year 5	10/26	0/1
School B	Year 8	3/4	3/4
Secondary C	Year 8	1/14	1/1
Secondary D	Year 9	2/23	0/1
Secondary E	Year 9	2/20	0/1
Secondary F	Year 10	9/24	unknown *
Secondary G	Year 11	1/9	1/1
**Total**		**28/120**	**5/9**

* Please note we use ‘unknown’ in the tables if the VIP student did not identify her/himself when s/he completed the questionnaire.

Our findings ( Table 5) show that only 21% of the overall sample of students and 33% of the visually impaired students thought diagrams would be easier to understand when they use small words to explain concepts. Thus, it could be argued that language accompanying the diagrams does not appear to present a barrier for the pupils.

**Table 5.** Students’ response to: “Diagrams in science are easy to understand when they use small words to explain things”

Class	Year level	Respondent students/Total number of students in class	Respondent VIP students/Total number of VIP students in class
Primary A	Year 5	9/26	1/1
School B	Year 8	1/4	1/4
Secondary C	Year 8	3/14	0/1
Secondary D	Year 9	2/23	0/1
Secondary E	Year 9	5/20	0/1
Secondary F	Year 10	3/24	unknown *
Secondary G	Year 11	2/9	1/1
**Total**		**25/120**	**3/9**

* Please note we use ‘unknown’ in the tables if the VIP student did not identify her/himself when s/he completed the questionnaire.

Just under a quarter (24%) of the sample indicated that diagrams are easy to understand when they are colourful ( Table 6). Just over 50% of the visually impaired sample indicated that diagrams were easy to understand when they are colourful. Only one student from School B did not support this view, probably because that student is unable to distinguish colours.

**Table 6.** Students’ response to: “Diagrams in science are easy to understand when they are colourful”

Class	Year level	Respondent students/Total number of students in class	Respondent VIP students/Total number of VIP students in class
Primary A	Year 5	5/26	1/1
School B	Year 8	1/4	1/4
Secondary C	Year 8	3/14	1/1
Secondary D	Year 9	6/23	1/1
Secondary E	Year 9	3/20	1/1
Secondary F	Year 10	7/24	unknown *
Secondary G	Year 11	4/9	0/1
**Total**		**29/120**	**5/9**

* Please note we use ‘unknown’ in the tables if the VIP student did not identify her/himself when s/he completed the questionnaire.

All the visually impaired students in School B felt that diagrams in science were easy to understand when they were not messy ( Table 7). But just under a third of the total sample (39/120) agreed that diagrams were easy to understand when they were not messy.

**Table 7.** Students’ response to: “Diagrams in science are easy to understand when they are not messy”

Class	Year level	Respondent students/Total number of students in class	Respondent VIP students/Total number of VIP students in class
Primary A	Year 5	15/26	0/1
School B	Year 8	4/4	4/4
Secondary C	Year 8	1/14	0/1
Secondary D	Year 9	6/23	0/1
Secondary E	Year 9	4/20	0/1
Secondary F	Year 10	9/24	unknown *
Secondary G	Year 11	0/9	0/1
**Total**		**39/120**	**4/9**

* Please note we use ‘unknown’ in the tables if the VIP student did not identify her/himself when s/he completed the questionnaire.

All of the students in the school for the visually impaired and 77% of year 5 (including the visually impaired student) found diagrams in science easy to understand when someone explained what they meant ( Table 8). Interestingly, the mainstream classes were mixed, ranging from 78% of year 11 students down to 29% of year 8 students, found it easy to understand diagrams if they were explained. Overall in the sample, 78% of the visually impaired students found it easy to understand diagrams when someone explained it to them, as did 53% of the total sample of students in the seven classes.

**Table 8.** Students’ response to: “Diagrams in science are easy to understand when someone explains what they mean”

Class	Year level	Respondent students/Total number of students in class	Respondent VIP students/Total number of VIP students in class
Primary A	Year 5	20/26	1/1
School B	Year 8	4/4	4/4
Secondary C	Year 8	4/14	0/1
Secondary D	Year 9	7/23	1/1
Secondary E	Year 9	9/20	0/1
Secondary F	Year 10	13/24	unknown *
Secondary G	Year 11	7/9	1/1
**Total**		**64/120**	**7/9**

* Please note we use ‘unknown’ in the tables if the VIP student did not identify her/himself when s/he completed the questionnaire.

Our findings ( Table 9) show that 25% of the overall sample of students found diagrams in science easy to understand when they are simple compared to nearly 50% of the visually impaired students in all seven classes.

**Table 9.** Students’ response to: “Diagrams in science are easy to understand when they are simple”

Class	Year level	Respondent students/Total number of students in class	Respondent VIP students/Total number of VIP students in class
Primary A	Year 5	7/26	1/1
School B	Year 8	2/4	2/4
Secondary C	Year 8	1/14	1/1
Secondary D	Year 9	3/23	0/1
Secondary E	Year 9	7/20	0/1
Secondary F	Year 10	8/24	unknown *
Secondary G	Year 11	2/9	0/1
**Total**		**30/120**	**4/9**

* Please note we use ‘unknown’ in the tables if the VIP student did not identify her/himself when s/he completed the questionnaire.

Over 50% of the visually impaired students (the majority of whom were from the school for visually impaired) found diagrams in science easy to understand if the same ones are used often. Only 14% of the total sample felt that repeated encounters with the same diagrams made those diagrams in science easy to understand ( Table 10).

**Table 10.** Students’ response to: “Diagrams in science are easy to understand when we use the same ones often”

Class	Year level	Respondent students/Total number of students in class	Respondent VIP students/Total number of VIP students in class
Primary A	Year 5	6/26	1/1
School B	Year 8	3/4	3/4
Secondary C	Year 8	1/14	1/1
Secondary D	Year 9	2/23	0/1
Secondary E	Year 9	2/20	0/1
Secondary F	Year 10	2/24	unknown *
Secondary G	Year 11	1/9	0/1
**Total**		**17/120**	**5/9**

* Please note we use ‘unknown’ in the tables if the VIP student did not identify her/himself when s/he completed the questionnaire.

Over 50% of the visually impaired students found diagrams in science easy to understand when they had seen or used them before ( Table 11). However, the majority of those were from the school for visually impaired students. Only 16% of the overall sample felt diagrams were easy to understand if they had seen them before.

**Table 11.** Students’ response to: “Diagrams in science are easy to understand when I’ve seen or used them before”

Class	Year level	Respondent students/Total number of students in class	Respondent VIP students/Total number of VIP students in class
Primary A	Year 5	9/26	1/1
School B	Year 8	2/4	2/4
Secondary C	Year 8	0/14	0/1
Secondary D	Year 9	1/23	1/1
Secondary E	Year 9	2/20	0/1
Secondary F	Year 10	4/24	unknown *
Secondary G	Year 11	1/9	1/1
**Total**		**19/120**	**5/9**

* Please note we use ‘unknown’ in the tables if the VIP student did not identify her/himself when s/he completed the questionnaire.

## Discussion

Our findings showed that in mainstream schools significant numbers of students found that diagrams were not easy to understand regardless of whether they were sighted or visually impaired. Our findings also showed that visually impaired students at the school for the visually impaired thought that diagrams in science were easy to understand. This may be because the classes were smaller in that school, and our observation of the classes (see McDonald & Rodrigues, 2016) showed that the strategies and skills used by the teachers were more individually focussed. However, visually impaired students at the school for the visually impaired found diagrams hard work and felt that they were more able to understand them if the diagrams were not messy. This could be for several reasons. Our observation showed that in the school for visually impaired students, the teacher ensured that the students engaged with the diagrams and that the students had to take responsibility for making sense of the information; in mainstream classes the classroom assistant engaged with the diagram and acted as a type of interpreter, translated their viewing into a verbal account for the relevant student. Another reason may stem from the fact that in the school for the visually impaired the teacher spent a great deal of time ensuring the whole class understood the diagrams, which made it ‘hard work’ given the effort required by the students to understand the diagrams. Another reason may arise from the fact that the students observed in the school for the visually impaired were congenitally blind, whereas the visually impaired students in mainstream classes were partially sighted and the majority had 1:1 learning support assistants who explained the diagrams and assisted in answering questions. This might also account for why the visually impaired students in the mainstream classes did not find the diagrams ‘hard work’, or even notice if they were messy! Talking specifically about special education Kumar *et al*. (2001) cited the work of Kamii & DeVries (1993) who argued a constructivist principle: that a teacher should not serve as a source of knowledge but should see their role as helping the student to construct their knowledge. During our project the teachers commented on the need to cover an increasing amount of subject content and identified the role of assessment in driving and determining goals. This may inadvertently mean that life skills such as learning how to interpret diagrams become overwhelmed by other goals.

As the teacher in the school for visually impaired students commented, sighted students can “flit” back and forth on the diagram, whereas for a visually impaired student who can only see or touch a small part of the diagram at a time to ”flit” would mean losing their place and orientation within the diagram. It can also be very confusing to a visually impaired person to read a tactile diagram where an element goes behind another element and then reappears. To a visually impaired person there are three entities not two. Not surprisingly then, reading a tactile or large print diagram requires a great deal of memory to hold a current interpretation and understand the position with regard to the complete diagram. This supports an argument for simplicity in diagrams. Sighted students incidentally learn how to interpret visual information from a young age. Students who acquire sight loss need to learn the different rules associated with reading tactile diagrams, or large print and those who are congenitally blind do not have visual memories to rely upon.

The fact that only 16% of the students in our convenience sample found that diagrams were easier to understand if they had encountered them before suggests that increased viewing does not result in better familiarity. Interestingly, in the school for the visually impaired the students did find that using the same diagrams often made the diagrams easier to understand. Access to assistance may also be a factor, for the two visually impaired students in mainstream classes who found using the same diagram often made the diagrams easier to understand did not have a 1:1 classroom assistant support.

In our sample of visually impaired students, a majority felt that colour would benefit their understanding of diagrams. The one visually impaired student, who did not support this view, has difficulty with colour. Thus it should be noted that one solution might not suit all visually impaired students. As Kevin Carey (Chair, Royal National Institute for the Blind group) said, “the root philosophical problem is the confusion between treating people equally and identically.”

## Data availability

The data referenced by this article are under copyright with the following copyright statement: Copyright: © 2016 McDonald C and Rodrigues S

All source data relevant to this study are presented in Table 1– Table 11.

## Supplementary material

Student Questionnaire

**1. Diagrams in science are:**

- **□ easy to understand**
- **□ boring to use**
- **□ exciting**
- **□ hard work**

**2. Diagrams in science are easy to understand when:**

- **□ they use small words to explain things**
- □ they are colourful
- **□ they are not messy**
- □ someone explains what they mean
- **□ they are simple**
- □ we use the same ones often
- **□ I’ve seen them before**

**3. If your friend found it hard to understand a diagram in science what would you do to make it easier to understand?**
