# Supplementary material for: Sighted and visually impaired students’ perspectives of illustrations, diagrams and drawings in school science
Source: Wellcome Open Res. 2016 Nov 15;1:8. [Version 1] doi: 10.12688/wellcomeopenres.9968.1 (PMC5133386; doi:10.12688/wellcomeopenres.9968.1)
Supplement: Peer review history

# Peer review: Referee response for version 1

**DOI:** 10.21956/wellcomeopenres.10743.r17574

**Published:** 2016-12-09

**Authors:** Jack Holbrook, Miia Rannikmae

**Affiliations:** 1 Centre for Science Education, University of Tartu, Tartu, Estonia

This research article is based on a simple questionnaire administered to students seeking perspectives on non-written text such as illustrations, diagrams, etc. The article is well written and easy to follow, leading to clear and justified conclusions.

 The title of the article is very appropriate and the abstracts provides a useful overview of the research. The study design is kept simple, but is very suitable for the intended research, well executed and the analysis, although limited, is very suitable for the intended investigation.

 Although the questionnaire items are very clear and appropriate, the limitation for the reader is in the appreciation of the difficulty difference in dealing with diagrams, etc. between well sighted and visual impaired students. It is suggested here that an example of such a diagram is helpful as well as insights into how the gaining of the holistic picture by sighted students is an advantage versus the building up of parts by visually impaired students (apart from the speed of operation which is well alluded to in the research).

 It is noted that the coverage of the discussion is not for a specific diagram, set of diagrams, or for a specific educational level of student. This means that it looking at the findings in a generalised sense and aspects such as validity of the diagram, or the reliability of responses are not entertained. In this respect the paper does not lead to how good diagrams are recognised or how developers of teaching materials need to pay attention to diagrams, charts, etc. 

 As a final point, the article is easy to read and could thus serve as a useful source for PhD students as they seek to build their own studies, especially non-native English readers.

We have read this submission. We believe that we have an appropriate level of expertise to confirm that it is of an acceptable scientific standard.

# Response

**Published:** 2016-12-09

**Authors:** Susan Rodrigues

**Affiliations:** Liverpool Hope university

Thank you for that comprehensive review of our article. Your feedback is appreciated more so given PISA findings show that Estonia appear to have done rather well in School Science!

 We take on board your comments regarding the challenge in appreciating the difficulty for sighted and visually impaired students in making sense of diagrams. We are hoping that our coverage of the research reported in this paper (drawing on the questionnaire) will be complemented by the papers/articles in production that draw on observation of classroom practice, taking into consideration working memory capacity, positioning theory and teacher and student agency in order to discuss specific diagrams, sets of diagrams, and specific student educational levels.

 We have also opted to provide guidance with regard to creating accessible diagrams based on our research findings, in the form of a short vodcast that is available at: https://www.youtube.com/watch?v=cmj0QXJA3Iw

# Peer review: Referee response for version 1

**DOI:** 10.21956/wellcomeopenres.10743.r18020

**Published:** 2016-11-28

**Authors:** Varughese K Varughese

**Affiliations:** 1 Department of Vocational Heath and Sciences, Royal Melbourne Institute of Technology, Melbourne, VIC, Australia

- The title is appropriate for the content of the article.
- The abstract and discussion are well documented.
- The study is described in enough detail to replicate it.
- Data collection was conducted with appropriate ethical approval.
- The result section describes the data and the Tables 1 to 11 are self-explanatory. However, one observation is that Table 10 & Table 11 depict more or less the same data. In my view this could confuse students and Table 11 could have been avoided altogether or changed to ‘science becomes boring when the same diagrams are repeatedly used’.
- The research article throws light on the measures to be taken for the enhancement of learning and teaching processes for visually impaired students in STEM subjects.
- The article depicts the challenges faced in catering for the need for visually impaired students.
- Proper referencing is done.

I have read this submission. I believe that I have an appropriate level of expertise to confirm that it is of an acceptable scientific standard.

# Response

**Published:** 2016-12-09

**Authors:** Susan Rodrigues

**Affiliations:** Liverpool Hope university

Thank you for the review of our article. Not sure that collapsing the statements that resulted in data we presented in table 10 and table 11 is the way to go! For changing them to ‘science becomes boring when the same diagrams are repeatedly used’, fails to account for other reasons why diagrams becomes boring! Also, what our findings suggest is that diagram familiarity does not necessarily make it easier to understand.

# Peer review: Referee response for version 1

**DOI:** 10.21956/wellcomeopenres.10743.r17573

**Published:** 2016-11-22

**Authors:** Neil Taylor

**Affiliations:** 1 School of Education and Social Work, University of Dundee, Dundee, UK

The title is appropriate and abstract provides a good summary of the article. The article is of importance to teachers involved in science education as it clearly demonstrates the challenges faced by sighted and visually impaired pupils as they try to interpret IDDs. The article also draws to the attention of practitioners the further challenges visually impaired learners face.  The article illustrates the ways in which pupils find interpreting IDDs difficult and indicating more effective ways for pupils to engage with IDDs.

 There are claims though on p4 which state that, "over 50% ...... did not find diagrams easy to understand".  My challenge to this claim is that the authors are including responses such as "boring to use" and "exciting" as part of the total not responding to "easy to understand".  Boring and exciting do not necessarily imply 'not easy to understand'.

 This a very accessible piece of work and will be of interest to practitioners.

I have read this submission. I believe that I have an appropriate level of expertise to confirm that it is of an acceptable scientific standard.

# Response

**Published:** 2016-11-22

**Authors:** Susan Rodrigues

**Affiliations:** Liverpool Hope university

Thank you for the review.

 We agree, boring and exciting do not necessarily imply easy to understand- that is why the data is reported in individual tables for each statement. So Table 1 on page 4 (and the percentage) only refers to the responses related to diagrams are easy to understand (it does not include responses to the other statements such as boring to use or exciting in that total). The responses to diagrams in science are boring are reported in Table 2 and the responses to diagrams in science are exciting are reported in Table 3. Thank you again for your review.

# Peer review: Referee response for version 1

**DOI:** 10.21956/wellcomeopenres.10743.r17576

**Published:** 2016-11-21

**Authors:** Norman Reid

**Affiliations:** 1 University of Glasgow, Glasgow, UK

The overall presentation is clear, with adequate referencing.

 I think ‘constructivist views’ should be stronger. The evidence that learners construct understandings idiosyncratically is overwhelming.  Perhaps replace ‘views’ by ‘findings’.

 In interpreting the findings, the key possibly rests in table 7. Working memory is overloaded when a diagram is too cluttered. There is considerable circumstantial evidence that diagrams can have a positive or negative value in understanding and that limitations of working memory explain the differences.  The work of Sweller (1999) might be worth considering ^1^.  This insight might strengthen the discussion section, which is largely descriptive.

I have read this submission. I believe that I have an appropriate level of expertise to confirm that it is of an acceptable scientific standard.

## References

[1] SwellerJ: Instructional design. *Australian Educational Review*.1999;

# Response

**Published:** 2016-11-22

**Authors:** Susan Rodrigues

**Affiliations:** Liverpool Hope university

Thank you for your review. We will certainly take on board the issue of working memory (a lack of working memory on our part). We will consider using Sweller's work and the research on working memory in future reports that may arise during the life time of this project. Thank you for your insight.
